# Influence of Ego Depletion on Individual Forgiveness in Different Interpersonal Offense Situations

**DOI:** 10.3389/fpsyg.2021.631466

**Published:** 2021-07-22

**Authors:** Yangen Zhou, Lihua Zhao, Yibo Yang, Xianmin Liu

**Affiliations:** ^1^Normal College, Changshu Institute of Technology, Suzhou, China; ^2^Taizhou College, Nanjing Normal University, Taizhou, China; ^3^School of Psychology, Nanjing Normal University, Nanjing, China

**Keywords:** forgiveness, ego depletion, offense situations, cognition, morality

## Abstract

Forgiveness, as an important content in the field of morality, means that the offended person overcomes the negative emotion, cognition, and behavior toward the offender and replaces it with positive emotion, cognition, and behavior. Based on the theory of the limitation of psychological resources, ego depletion (ED) will lead to the weakening of self-regulation function, thus making some immoral behaviors, which is not conducive to individual forgiveness. In order to explore the influence of ED on individual forgiveness in different interpersonal offense situations, this study used the Stroop task to manipulate the level of ED and used imaginary situations to distinguish offending situations. We found that the level of forgiveness in a serious offense situation was significantly lower than that in a mild offense situation, *p* < 0.001, partial η^2^ = 0.158. In different interpersonal offense situations, ED has different effects on forgiveness. In the severe offense situation, the forgiveness level of high-ED individuals was significantly lower than that of the low-ED individuals, *p* = 0.023, partial η^2^ = 0.144; in the mild offense situation, the forgiveness level of high-ED individuals was significantly higher than that of low-ED individuals, *p* = 0.029, partial η^2^ = 0.140. The results showed that different levels of ED have no consistent effect on forgiveness in different interpersonal offense situations; high ED hinders individual forgiveness in serious offense situations but can promote individual forgiveness in mild offense situations.

## Introduction

In real life, interpersonal offenses occur from time to time. The occurrence of offenses and the coping strategies of both sides are not only interpersonal problems but also moral problems. For example, forgiveness requires the offended to reduce revenge motivation, increase benevolence motivation, and replace negative feelings with positive emotions (Enright et al., 1992; Hargrave and Sells, 1997; McCullough et al., 1998; Carnevale and Fujita, 2016; Hagger and Chatzisarantis, 2016; Forster et al., 2019; see also for a review, Bertrams, 2020). As a sub-component of benevolence, forgiveness is an important content in the field of morality. Actually, forgiveness is mainly defined as a process of transformation and change, that is, the change of the victim’s attitude toward the offender or pro-social motivation, from negative to positive emotion, cognition, and behavior (Enright et al., 1992; McCullough, 1998). Several researchers have designated forgiveness as a continuum of prosocial changes from hostility to friendliness (e.g., Forster et al., 2019). There was evidence that forgiveness is an attitude or emotion that the victim no longer hates the offender (Hargrave and Sells, 1997). In this study, we regard forgiveness as a process of prosocial motivation transformation, that is, overcoming the negative emotion, cognition, and behavior of the offender, and replacing it with positive emotion, cognition, and behavior (Enright et al., 1992; McCullough et al., 1998).

Forgiveness can not only relieve anger, anxiety, depression, and other negative emotions, but also promote the generation of positive emotions and improve life satisfaction (Friedberg et al., 2007). It is not only closely related to the mental health of an individual, but also to the physical health of an individual. For example, forgiveness can relieve facial EMG tension, reduce individual heart rate and blood pressure, accelerate the speed of cardiovascular recovery, and assist in the treatment of physiological diseases (Witvliet et al., 2008). In recent years, the influencing factors of forgiveness were investigated from the aspects of an offending situation and apology, personal characteristics, individual cognition, and cultural environment. However, few investigations pay attention to the influence of ego depletion (ED) on forgiveness.

Ego depletion is a concept based on the theory of limited psychological resources. Based on Freud’s theory of personality structure, Baumeister et al. defined the ED as the decrease of the ability or willingness of self-participation in volitional action due to the previous exercise of will. From this point of view, any self-regulation behavior, whether it is the need to set appropriate goals, plan, implement goal-oriented behavior, evaluate the goal progress of a person, or resist the temptation to promote the implementation of long-term goals, should cause ED (Baumeister et al., 1998). Based on self-regulation (trying to control or change their own response), Muraven et al. (1998) proposed a narrow concept of ED, which suggested that when people adjust themselves, they should show a decline in other tasks that may need self-regulation. By the meta-analysis of ED studies, Hagger et al. (2010) defined ED as a state in which individuals consume self-control resources in self-control activities, which leads to temporary impairment of executive function. More recently, Carnevale and Fujita (2016) pointed out that ED can be defined as a significant decrease in the ability or willingness to self-regulate thoughts, emotions, and behaviors after some initial behaviors.

So far, there has been a debate about the nature and stability of ED (see for a review, Bertrams, 2020). Regarding the nature of ED, some researchers have found that glucose supplementation, cognitive control, and emotional relief can alleviate ED, and hence, they have questioned the nature of ED, that is, whether ED and fatigue are the same processes (Sander et al., 2012). Moreover, although Hagger et al. (2010) found that there was a moderate effect size of ED (*d* = 0.62), indicating the objectivity of ED effect, after controlling for the influence of publisher bias, Carter and McCullough (2014) found that effect size in Hagger et al.’s study Hagger et al. (2010) was significantly reduced or even disappeared, suggesting that ED effect may not exist. On the contrary, in the large sample study, a certain degree of ED effect was observed after controlling for the influence of publisher bias (Tuk et al., 2015). Although the nature and difficulty of the task generally affect the emergence of ED effect, the absence of ED effect in the multilaboratory repetitive study conducted by Hagger and Chatzisarantis (2016) could be due to the difficulty of the task significantly lower than that of the study by Baumeister et al. (1998). Controlling the modulation of small size and using usually difficult items from the GRE test, the meta-analysis showed the stable ED effect (Carter et al., 2015).

Converging evidence showed that ED has a great influence on cognition, emotion, and behavior. In terms of cognition, ED affects the social cognitive process of an individual and promotes impulsive decision making; in terms of emotion, the empathetic nature of individuals after ED is poor, and it is difficult to suppress anger (Tangney et al., 2004); in terms of behavior, ED significantly reduces the self-control of an individual and increases the bad behavior (Wang et al., 2017). A large number of studies have proved that ED has a negative impact on moral behavior. After ED, positive emotions of an individual toward others are reduced, and positive behaviors, such as emotional control, impulsive behavior, risky behavior, aggressive behavior, and immoral behavior, have been reduced (DeWall et al., 2008; Mead et al., 2009; Barlett et al., 2016). Forgiveness requires individuals to suppress their negative impulses and produce positive cognition, emotion, and behavior toward the offender. It can be concluded that in the case of ED, the level of forgiveness after being offended will be reduced. At present, there is not much research on the relationship between ED and forgiveness, especially the direct research on the relationship between them. Some researchers have examined the relationship between ED and aggressive behavior after being challenged. It is found that in the face of provocation (offensive events), individuals with ED are more likely to engage in aggressive behaviors (Barlett et al., 2016). It can be considered that after the offense, the individuals with ED prefer not to forgive. In addition, Balliet and Joireman (2010) tested the effectiveness of the compensatory model of forgiveness with four studies, that is, forgiveness requires a high degree of care for others or a high degree of self-control, which proves that individuals with high ED are less likely to forgive others.

In addition, some studies have found that forgiveness in lovers is affected by both ED and offending situations, and the influence of ED on forgiveness is inconsistent in different degrees of the offending situation. It is found that when individuals are confronted with offense by their partner, individuals in ego deprivation state are more difficult to forgive severe offenses (Pronk et al., 2010), while it is interesting that they are more likely to forgive minor offenses (Stanton and Finkel, 2012), and this interaction is regulated by the offense severity. This reminds us that it is necessary to consider the severity of the offense situation when exploring the relationship between ego redemption and forgiveness.

This study will explore the influence of ED on forgiveness, which will not only provide a new theoretical perspective for the influencing factors of forgiveness but also enrich the theoretical research on ED and forgiveness. In order to study the influence of different levels of ED on forgiveness, it is necessary to consider the factor of different offending situations. If ED can predict forgiveness level, it will be predicted that the forgiveness level of individuals with high-level ED is significantly lower than that of individuals with low-level ED; If the offense situation affects the forgiveness level of an individual, the level of forgiveness is significantly lower in the individual with serious offense situations than that with minor offense situations. Moreover, the interaction between ED and an offense situation could be also found.

## Materials and Methods

### Participants

One hundred and twenty participants (68 women and 52 men; mean age: 23.06 ± 2.41 years) were randomly recruited at Nanjing Normal University of China, with homogeneous variables critical to the study. Participants with a history of neurological and psychiatric disorders, who were receiving psychoactive drug therapy that could change cognitive ability, were excluded from the study. All participants had a normal or corrected-to-normal vision and normal hearing. The study was approved by the local ethics committee of Nanjing Normal University and all participants gave their written informed consent for the study and were paid for their participation.

### Stimuli and Procedure

#### Stroop Consumption Task

In this task, “red,” “yellow,” “blue,” and “green” were written in four colors: red, yellow, blue, and green. Then, by controlling the proportion of consistent and inconsistent color words in the Stroop task, participants had different degrees of ED (Gailliot et al., 2007; Lei, 2017; Zhou, 2017). According to the different experimental conditions, the high-ED group completed 120 stimuli (high ED stimuli), 24 stimuli with consistent color words, and 96 stimuli with inconsistent color words; the low-ED group completed 120 stimuli (low-ED stimuli), 96 stimuli with consistent color words, and 24 stimuli with inconsistent color words. After completing the Stroop task, the participants were asked three ED tests, such as “how much effort did you put into resisting the influence of the meaning of words on the color of words?,” using the seven-point scale (1 means you do not need to put in any energy and 7 means you put in all your energy) (Dou et al., 2014).

#### The Manipulation of Offending Situations and the Measurement of Forgiveness

Participants in the high ED read three serious imaginary situations and participants in the low ED read three mild imaginary situations (Zhou, 2017): (1) serious offense is obviously harmful. For example, you and your classmates make an appointment to climb the mountain at the weekend and get together at the top of the mountain. Unfortunately, it rained that day. They temporarily canceled the plan and did not inform you when they wanted to make fun of you. Therefore, you still went there. However, due to the wet and slippery road on rainy days, you accidentally stepped on the mountain and fell down and broke your arm; (2) mild offense is slightly injurious. Consistent with the previous content of the severe situation, the result is that because of the heavy rain, you get wet and go home with a cold. Participants were asked to imagine each scene as vividly as possible. After each offense was conceived, participants completed a test to assess perceived severity and a test to evaluate forgiveness (the sum of the scores of positive cognition, emotion, and behavior of the student is taken as the score of forgiveness), with 9 points for each test (Dou et al., 2014; Lei, 2017; Zhou, 2017). For example, a mild offense (in Chinese) was “You and your classmates made an appointment to climb the mountain at the weekend and get together at the top of the mountain. Unfortunately, it rained that day. They temporarily canceled the plan and did not inform you as they wanted to play a joke with you. So you still went. Because of the heavy rain, you caught a cold after you got wet and went home” and there were 5 questions to answer, (1) how much harm do you think you’ve been hurt? (1, low damage; 9, high damage); (2) how much do you think you forgive your classmates? (1, don’t forgive; 9, forgive); (3) looking back on the whole event, what’s your mood like? (1, hate; 9, don’t hate); (4) what do you think of the moral character of your classmates? (1, bad character; 9, good character); (5) if your classmates are in trouble, what will you do? (1, don’t help; 9, help). In this example, the first question examines the degree of offense, the second question tests forgiveness level, and questions 3–5, according to the connotation of forgiveness, measure the level of forgiveness from the aspects of emotion, cognition, and behavior. The sum of 3–5 questions is forgiveness score (e.g., Zhou, 2017).

### Data Analysis

The 120 participants were randomly assigned to the high-ED group or the low-ED group to complete the above Stroop task and fill in the operation check questions of ED. Before completing the Stoop task, the ED test did not actually differ between the two groups. Then, they were asked to immediately read the randomly assigned interpersonal offense situations, imagine, and complete the follow-up measurements. The response time and accuracy as well as the forgiveness score were recorded and submitted to a two-way ANOVA with an offensive situation as a within-subject factor (serious and minor) and ED as a between-subject factor (high and low). Using the Greenhouse–Geisser epsilon correction factor, we corrected degrees of freedom.

## Results

### Performance Test

**FIGURE 1:**
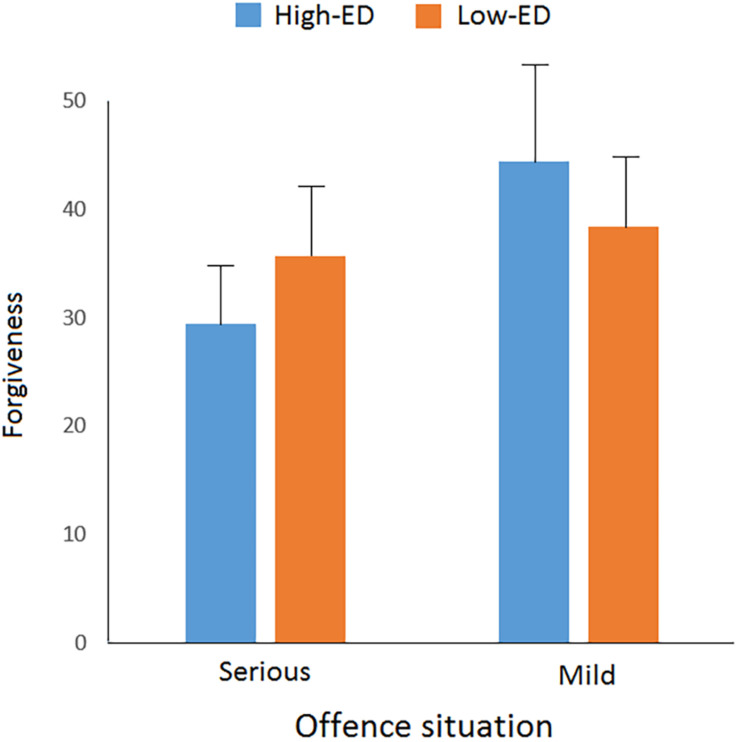
Forgiveness scores in different offense situations for high-ego depletion (ED) and low-ED groups, respectively.

After completing Stroop task, the independent sample *t*-test showed that the high-ED group reported higher ED than the low-ED group, *t*(118) = 3.665, *p* < 0.001, *d* = 0.473 (Figure 1). Among them, they felt more tired [*t*(118) = 2.678, *p* = 0.008, *d* = 0.346], put in more energy [*t*(118) = 2.581, *p* = 0.011, *d* = 0.333], and felt more energy loss [*t*(118) = 3.917, *p* < 0.001, *d* = 0.506]. In addition, the reaction time was longer [*t*(118) = 3.020, *p* = 0.003, *d* = 0.390]. These data showed that the proportion control of consistent and inconsistent color words in the present Stroop task effectively manipulated the degree of ED.

For the test of offensive situation, the independent sample *t*-test showed that the participants in the serious offense situation were more hurt than those in the mild offense situation, *t*(118) = 2.407, *p* = 0.018, *d* = 0.311, revealing that the present different interpersonal offense situations were effectively manipulated.

### Analysis of Forgiveness Dimension

Forgiveness level consists of positive cognition, positive emotion, and positive behavior toward the offender. As shown in Table 1, positive cognition, positive emotion, and positive behavior were significantly positively correlated with forgiveness; positive cognition, positive behavior, positive emotion, and forgiveness were significantly positively correlated with the level of forgiveness. The results verified the validity of the forgiveness score in this study, and also an effective verification of the connotation of forgiveness.

**TABLE 1:** Mean value, SD, and correlation coefficient of variables.

variable	M	SD	Positive cognition	Positive emotion	Positive behavior	Forgiveness	Forgiveness level
Positive cognition	11.117	3.697	1				
Positive emotion	11.067	4.204	0.685**	1			
Positive behavior	14.875	5.518	0.645**	0.593**	1		
Forgiveness	36.975	11.583	0.859**	0.853**	0.888**	1	
Forgiveness level	12.450	4.290	0.692**	0.651**	0.659**	0.769**	1

**p < 0.01.

### The Influence of ED and an Offending Situation on Forgiveness

Ego depletion and an offending situation were used as independent variables and forgiveness as dependent variables. The main effect of ED was not significant, *F*(1, 116) < 1, but the main effect of an offensive situation was significant, *F*(1, 116) = 21.772, *p* < 0.001, partial η^2^ = 0.158. Importantly, we found significant two-way interactions between ED and an offensive situation, *F*(1, 116) = 10.175, *p* = 0.002, partial η^2^ = 0.181. Further analysis revealed that the forgiveness score of the high-ED group was significantly lower (29.5 ± 9.39) than that of the low-ED group (35.6 ± 9.63) in a severe offense situation, *F*(1, 116) = 5.286, *p* = 0.023, partial η^2^ = 0.144, whereas in a mild offense situation, forgiveness score of the high-ED group was significantly higher (44.4 ± 11.46) than that of the low-ED group (38.4 ± 10.94), *F*(1, 116) = 4.893, *p* = 0.029, partial η^2^ = 0.140.

## Discussion

The purpose of this study was to explore the effect of ED on forgiveness, and to examine the different effects of ED on forgiveness in different interpersonal offense situations. In this study, the Stroop task was used to manipulate the degree of ED in the laboratory context, and the level of aggression was manipulated by imagining the situation and substituting the subject into the offending situation. Stroop task is the most commonly used paradigm in the study of ED (e.g., Gailliot et al., 2007; Dou et al., 2014; Lei, 2017; Zhou, 2017). Imagining the offending situation can help us to standardize the objective severity of the offense. In the past, the questionnaire method was used to make the subjects recall the offending situation. Although the ecological validity was good, the operation control was insufficient. The method of recalling the situation was more likely to arouse the emotion of the subject and may cause discomfort. Therefore, the use of imaginative situations in this study is complementary to previous studies to a certain extent.

The present results showed that the main effect of ED on forgiveness was not significant, whereas the main effect of an offending situation on forgiveness was very significant, revealing that the level of forgiveness in a mild offense situation was significantly higher than that in a serious offense situation. The interaction effect of ED and an offending situation on forgiveness was also very significant, showing that in the serious offense situation, the higher the degree of ED, the lower the level of forgiveness, however, in the mild offense situation, the higher the degree of ED, the higher the level of forgiveness.

Consistent with the results of this study in the context of a serious offense, many studies supported that ED can reduce the level of forgiveness (Balliet and Joireman, 2010; Barlett et al., 2016). However, these studies classified ED with or without ED and did not consider the situational factor of the offending situation. Although some studies have shown that ED was associated with negative interpersonal outcomes, a few studies have shown that it sometimes leads to pro-social behavior (Apfelbaum and Sommers, 2009). This study confirmed that ED can sometimes promote beneficial relationship processes and outcomes. Consistent with the results in intimate relationships, in general, interpersonal relationships, the degree of ED and an offending situation also have an interactive effect on forgiveness. In the situation of a serious offense, high ED hinders individual forgiveness; in a mild offense situation, high ED promotes individual forgiveness.

Several explanations could account for the present fact that individuals with high ED are more likely to forgive minor offenses. The first is that the higher the ED is, the less contemplation of offense is. Therefore, compared with low-ED individuals, individuals with high ED may have less negative cognition of offensive behavior. However, when the degree of ED is not high, people still have enough self-regulation resources. They may over-analyze the offense and convince themselves that forgiveness is unnecessary. Therefore, in the situation of slight offense, high-attrition individuals are more likely to forgive than low-attrition individuals, which may be because they are too tired, but they are not willing to waste energy on such trivial matters because of the minor offense. Second, Worthington et al. (2001) believe that individual self-control may be related to forgiveness by suppressing destructive impulses or regulating negative emotions. One way for people with strong self-control or self-interest to suppress revenge motives is to think about the value of others to help them (Fitzsimons and Shah, 2009). In line with this hypothesis, some studies have found that in intimate relationships, egoists have stronger relationship commitment and willingness to sacrifice than Pro socialists (Van Lange et al., 1997). Thus, combined with Fitzsimons and Shah (2009), it is possible that egoists can be motivated to cooperate when it is in the long-term interests of individuals. Therefore, individuals with a high degree of ED will make a decision of forgiveness after a simple measurement of the harm and the long-term interests after being slightly offended; while the individuals with a low degree of ED will think more about the current loss and long-term interests after being slightly offended, but will make a more cautious decision of forgiveness. Actually, egoism could be an incentive factor for forgiveness, and hence, for the sake of long-term self-interest, even if the degree of ED is relatively high, individuals still have a higher willingness to forgive others in the situation of slight offense. Obviously, this issue needs further investigation.

It is widely accepted that self-regulation consists of two stages, one is to suppress destructive impulses and the other is to participate in constructive impulses, both of which require independent self-control. There was evidence that severe ED may impair the ability of a person to participate in both stages of self-regulation, whereas slight ED may only affect the ability of the second stage (Finkel and Campbell, 2001). Although individuals with mild ED have self-regulation resources to suppress destructive impulse, they are not enough to engage in constructive behavior, because they have enough resources to engage in cognitive reassessment process, so they have not low forgiveness for a serious offense, but not high forgiveness for a minor offense; while individuals with high ED have impaired both stages of the regulation process, so they have less reflection on offense. Less, more extreme in judgment and response, this reduced tendency to contemplate seems to be the reason for the polarization of ED to individual forgiveness in different offending situations.

Finally, the “sticking anchor” hypothesis may also provide an explanation for the results of this study. ED weakens central executive control and increases sensitivity to salient cues. In other words, ED enhances the influence of external cues (anchors) on behavior (Banker et al., 2017). Pitesa et al. (2013) found that when clues of a person about the impact of a certain behavior on interpersonal relationship become obvious, subjects with ED will engage in behaviors that are more in line with social expectations. In other words, ED may lead people to rely more on external cues in the absence of strong internal impulses. Individuals with high ED are more likely to process confirmatory information, and they are less likely to actively infer (Schmeichel et al., 2003; Fischer et al., 2008), and their behavior is increasingly guided by instantaneous and situational factors. That is, obvious cues or stimuli have a greater impact on them than logically or ideally. In the context of this experiment, individuals with high ED were more likely to be affected by offending situations than individuals with slight attrition, so they were less forgiving in serious offenses and more forgiving in minor offenses.

It is noteworthy that in this study, according to Baumeister’s framework (Baumeister et al., 1998; Baumeister et al., 2007), ED is regarded as an individual’s consumption of psychological resources in the previous task for self-control, thus showing a temporary low-control state in the subsequent task. In future studies on ED, it would be necessary to first explore the stability of ED and clarify the conditions of ED effect, such as the task difficulty, type, and sample size. Second, the mechanism of psychological resources should be determined differently from aversion, opportunity cost, glycogen, and psychological fatigue. Finally, we should consider the budget and dynamic calculation of the event because their combination may be an effective way to predict the individual ED.

## Conclusion

This study explored the influence of ED on individual forgiveness level in different interpersonal offensive situations using the Stroop task to distinguish the offensive situations. We found that the level of forgiveness in a serious offense situation was significantly lower than that in a mild offense situation. In different interpersonal offense situations, ED has different effects on forgiveness. In the severe offense situation, the forgiveness level of high ED individuals was significantly lower than that of the low-ED individuals; in the mild offense situation, the forgiveness level of high-ED individuals was significantly higher than that of the low-ED individuals. The results showed that different levels of ED have no consistent effect on forgiveness in different interpersonal offense situations; high-ED hinders individual forgiveness in serious offense situations but can promote individual forgiveness in mild offense situations.

## Data Availability Statement

The original contributions presented in the study are included in the article/supplementary material, further inquiries can be directed to the corresponding author/s.

## Ethics Statement

The studies involving human participants were reviewed and approved by Nanjing Normal University. The patients/participants provided their written informed consent to participate in this study.

## Author Contributions

YZ and YY: data collection and the draft. LZ and YY: design and revised the manuscript. All authors contributed to the article and approved the submitted version.

## Conflict of Interest

The authors declare that the research was conducted in the absence of any commercial or financial relationships that could be construed as a potential conflict of interest.
